# Stimulation of Angiotensin II Type 2 Receptor Modulates Pro-Inflammatory Response in Microglia and Macrophages: Therapeutic Implications for the Treatment of Stroke

**DOI:** 10.3390/life13061274

**Published:** 2023-05-29

**Authors:** Abdulkarim Alshammari, Yohan Han, Timothy W. Jones, Bindu Pillai, Duo Zhang, Adviye Ergul, Payaningal R. Somanath, Susan C. Fagan

**Affiliations:** 1 Clinical and Experimental Therapeutics, College of Pharmacy, University of Georgia, Augusta, GA 30602, USA; 2 Charlie Norwood VA Medical Center, Augusta, GA 30904, USA; 3 Department of Clinical Pharmacy, Faculty of Pharmacy, Northern Border University, Rafha 76313, Saudi Arabia; 4 Vascular Biology Center, Augusta University, Augusta, GA 30912, USA; 5 Department of Pathology and Laboratory Medicine, Medical University of South Carolina, Charleston, SC 29425, USA; 6 Ralph H. Johnson VA Health Care System, Charleston, SC 29401, USA

**Keywords:** stroke, neuroinflammatory response, Compound **21**, renin–angiotensin system, BDNF, GDNF

## Abstract

Background: Sustained microglial activation contributes to the development of post-stroke cognitive impairment (PSCI). Compound **21** (C21), an angiotensin II type 2 receptor agonist, has shown some neurovascular protection after stroke. This study aimed to investigate the direct anti-inflammatory effects of C21 on macrophages, as well as brain innate immune cells. Methods: Murine microglial cell line (C8-B4) and RAW 264.7 macrophages were exposed to lipopolysaccharide (LPS) and co-treated with C21. Pro-inflammatory mediators were assessed via RT-qPCR and ELISA. Cellular reactive oxygen species (ROS) were evaluated via CellROXGreen staining, and nitrate production was assessed using Griess assay. Results: C21 suppressed LPS-induced inflammation and ROS generation in both cells. In microglia, C21 blunted LPS-induced mRNA expression of IL-1β, IL-12b, COX-1, iNOS, and IL-6. A similar pattern was observed in macrophages, where C21 suppressed LPS-induced IL-1β, TNF-α, and CXCL1 expression. These anti-inflammatory effects in microglia and macrophages were associated with increased neuroprotective gene expression, including GDNF and BDNF, in a dose-dependent manner. Conclusions: Our findings suggest a protective effect of C21 against the inflammatory response, in both macrophages and microglia, via suppression of the release of pro-inflammatory cytokines/chemokines and the generation of ROS while stimulating the production of neurotrophic factors.

## 1. Introduction

Over the past two decades, our knowledge of stroke pathophysiology has dramatically expanded, specifically, to include the role of the immune system combined with a more advanced understanding of the neurovascular unit. Neuroinflammation is known to be a secondary injury following ischemic stroke and results in long-term consequences, such as post-stroke cognitive impairment (PSCI). The injury after an ischemic stroke is mediated through a dynamic interaction between all the neurovascular unit components, including neuronal, glial, and vascular cells [1].

Microglia, the resident innate immune cells, are a key player in neuroinflammatory response after stroke [2]. Typically, in the brain, the primary role of resident microglia is to survey and maintain homeostasis [3]. In response to ischemic injury, microglial activation goes through dynamic functional changes throughout the neuro-inflammatory response [4]. Shortly after stroke, microglia are expected to adopt an anti-inflammatory phenotype, facilitating enhanced phagocytosis, fewer inflammatory mediators, and improved neuronal survival [4]. However, the anti-inflammatory status is transitory; shortly after, pro-inflammatory microglia begin dominating the injured area [4].

The pro-inflammatory microglia are characterized by less phagocytosis and higher production of pro-inflammatory cytokines and, ultimately, start a cascade of events leading to disruption of the blood–brain barrier, degradation of the extracellular matrix, and triggering of the infiltration of peripheral leukocytes into the central nervous system [4,5]. The recruitment of monocyte-derived macrophages (MDM) to the site of injury has a crucial role in the repair processes [6,7]. MDMs are classified into two major sub-populations: the pro-inflammatory and anti-inflammatory monocytes (designated as M1 and M1 phenotypes) [8]. Classically, MDMs were believed to mediate detrimental effects after stroke [9]; recent studies demonstrated that monocytes promote protective effects after stroke, likely through enhancing delayed-anti-inflammatory effects [10,11,12].

The brain renin–angiotensin system (RAS) is believed to be involved in the pathogenesis of stroke [13,14,15,16,17,18,19]. Preclinical data showed a direct correlation between angiotensin II (Ang II), the active neuropeptide in the renin–angiotensin system (RAS), and the severity of ischemic injury after stroke [20]. Several studies have shown that stimulation of the Angiotensin II type 2 receptor (AT2R), either indirectly via candesartan, an Angiotensin II type 1 receptor blocker, or directly by Compound **21** (C21), provided acute and long-term neurovascular protection and improved sensorimotor and cognitive outcomes after stroke in young, aged, hypertensive, and diabetes models [14,17,19,21,22]. These effects, in part, were mediated by regulating the neuroinflammatory response [22,23,24]. In the current study, we sought to further confirm the ability of C21 to mediate anti-inflammatory and neurotrophic effects in mouse C8-B4 microglial cell lines and RAW 264.7 macrophages.

## 2. Materials and Methods

### 2.1. Cell Maintenance, Culture, and Treatment Conditions

C8-B4 mouse microglial cell line and RAW 264.7 cells were obtained from American Type Culture Collection (ATCC, Manassas, VA, USA) and were cultured in DMEM media, supplemented with 10% heat-inactivated fetal bovine serum and 1% Penicillin–Streptomycin (ATCC, Manassas, VA, USA) at 37 °C in a humidified 5% CO_2_ incubator. Cells were seeded at a density of 1–1.5 × 10^6^ in 60 mm dishes for mRNA and protein expression experiments, 10–12 × 10^4^ in 96-well plates for MTS viability assays, 1 × 10^4^ in 6-well plates for trypan blue staining, and 1 × 10^4^ in 60 mm dishes for ROS generation experiments. For all experiments, cells were allowed to adhere for 24 h and then maintained in a serum-free condition overnight before any treatment. C8-B4 and RAW 264.7 cells were treated with lipopolysaccharide (LPS) (100 ng/mL) to induce a pro-inflammatory response and co-treated with either different doses of C21 or vehicle for a total of 24 h [25].

### 2.2. RNA Isolation, cDNA Reverse Transcription, and Quantitative Real-Time rt-PCR

RT-qPCR was performed to measure the expression of pro-inflammatory and neurotrophic genes. Briefly, total RNA from cells was extracted using TRIzol™ reagent (Invitrogen, Carlsbad, CA, USA). The quantity and quality of isolated RNA were measured and assessed using Nanodrop (NanoDrop Technologies, Wilmington, DE, USA). An equal amount of RNA (500 ng) from each sample was reverse transcribed into complementary DNA (cDNA) using High-Capacity cDNA Reverse Transcription Kit (Applied Biosystems, Foster City, CA, USA). Quantitative RT-PCR was performed by StepOnePlus™ Real-Time PCR System, using SYBR Green Master Mix. The expression of the housekeeping gene, PPIA, was used as an endogenous control. The amplification condition was as follows: initial pre-incubation at 95 °C for 10 min, followed by amplification of the target DNA for 40 cycles (95 °C for 15 s followed by 60 °C for 1 min). The primers used in this study and their sequences are listed in Table S1.

### 2.3. ELISA

ELISA kits for pro-inflammatory cytokines, IL-1β (ab197742), TNF-α (ab208348) and CXCL1 (ab216951), were purchased from Abcam (Cambridge, UK), and all the analyses were performed according to the manufacturer’s protocol. Following treatment conditions, cell supernatants were collected and centrifuged at 2000× *g* × 10 min for complete removal of cell debris and then used to assess the secreted cytokines/chemokines using SpectraMax Multi-Mode Microplate Reader (Molecular Devices, San Jose, CA, USA). Standard curve plots of IL-1β, TNF-α, and CXCL1 ELISA are provided in Figure S1.

### 2.4. MTS Viability Assay

MTS viability assay (Promega, Madison, WI, USA) was used to determine the safety profile of C21. Briefly, 1 × 10^4^ cells were seeded into 96-well plates and allowed to adhere, and then cells were cultured in a serum-free medium overnight. Cells were treated with different concentrations of C21 (1 µM, 10 µM, 20 µM, 50 µM, and 100 µM) for a total of 24 h and then incubated with MTS reagent for ≈3 h at 37 °C. Absorbance was recorded at a wavelength of 490 nm using a microplate reader Agilent (Bio-TEK, Santa Clara, CA, USA). Accordingly, the percentage of cell viability was calculated based on the OD readings, using the following equation: % Cell viability= (OD/Mean OD of control) × 100.

### 2.5. Trypan Blue Staining: Viability Analysis

Cell viability was assessed via trypan blue staining. C8-B4 cells were seeded at a density of 1 × 10^5^ in 6-well plates and left to adhere for 24 h. Afterward, cells went through overnight serum starvation and were then treated with different doses of C21 (1 µM, 10 µM, 20 µM, 50 µM, and 100 µM) or vehicle for a total of 24 h. At this point, cells were trypsinized using 0.25% Trypsin and resuspended with PBS. Cell suspensions were mixed with 0.4% trypan blue solution (1:1 dilution) and allowed to stand for 2–3 min at room temperature. A 10 μL volume of cell suspension/trypan blue mixture was used to count viable/non-viable cells using TC20 automated cell counter (Bio-Rad, Hercules, CA, USA).

### 2.6. Cellular ROS Generation

Cellular production of reactive oxygen species was detected using the CellROX™ Green Reagent (Invitrogen, Waltham, MA, USA). Cells were seeded at a density of 1 × 10^6^ and then treated according to our experimental design. Briefly, cells were induced with LPS (100 ng/mL) and co-treated with either different concentrations of C21 (1 µM, 10 µM, 20 µM, and 50 µM) or vehicle for 24 h. Then, cells were washed with PBS and incubated with 5 µM CellROXGreen Reagent for 30 min at 37 °C. Fluorescence images were obtained using a Zeiss Observer Z1 microscope (Carl Zeiss, Oberkochen, Baden-Württemberg, Germany).

### 2.7. Nitrite and Nitrate Measurement

The level of nitric oxide was quantified using a Griess assay kit (Invitrogen, Waltham, MA, USA). Cell culture supernatant was collected from LPS-induced and C21-treated cells. Cells were centrifuged to eliminate cell debris, mixed with Griess reagent, and allowed to stand for approximately 30 min at room temperature. After 30 min, absorbance was recorded at 548 nm using a microplate reader (Bio-TEK). Sodium nitrate was used as a reference to detect the level of nitrate.

### 2.8. Statistical Analysis

Statistical analysis was performed using GraphPad Prism software. Multiple groups analysis (three or more) was performed using one-way ANOVA with either Dunnett’s multiple comparisons test for variables with normal distributions, or by pairwise comparisons using Mann–Whitney U tests for variables without normal distributions. Data distribution was evaluated via the Shapiro–Wilk test. Data are shown as mean ± SD, and a *p*-value of ≤0.05 was considered to be statistically significant.

## 3. Results

### 3.1. Compound **21** Demonstrates Acceptable Cytotoxicity at Lower Concentrations

**Figure 1:**
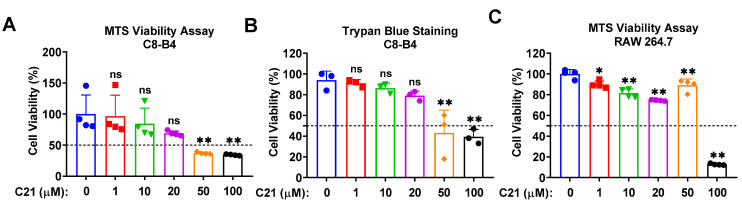
Dose-dependent effect of Compound **21** on C8-B4 microglia and RAW 264.7 macrophages demonstrate acceptable cytotoxicity at lower concentrations. The effect of different concentrations of C21 on cell viability was assessed via MTS (a tetrazolium salt that is reduced to purple formazan crystals) viability assay and trypan blue staining. C8-B4 microglia and RAW 264.7 macrophages were seeded into 96-well plates and treated with different concentrations of C21 (1 µM, 10 µM, 20 µM, 50 µM, and 100 µM) for a total of 24 h. Then, cells were incubated with MTS reagent for ~3 h at 37 °C. Absorbance was measured at a wavelength of 490 nm and the percentage of cell survival was calculated accordingly. C8-B4 cell proliferation was assessed using MTS assay (**A**), and trypan blue staining (**B**). Cell viability was assessed in RAW 264.7 macrophages via MTS viability assay (**C**). Values are the mean ± SD of 3–4 independent experiments analyzed by one-way ANOVA. ^ns^ *p* > 0.05, * *p* < 0.05, ** *p* < 0.01.

The safety of Compound **21** in microglia/macrophages was evaluated via MTS viability assay and trypan blue staining. Different concentrations of C21 (1 µM, 10 µM, 20 µM, 50 µM, and 100 µM) were evaluated, and our data demonstrate an acceptable safety profile up to 20 µM in C8-B4 microglia (Figure 1A,B), as well as in macrophages (Figure 1C).

### 3.2. Compound **21** Exhibits an Anti-Inflammatory Response in Microglia via Decreasing the Expression of LPS-Induced Pro-Inflammatory Cytokines/Chemokines in a Dose-Dependent Manner

**Figure 2:**
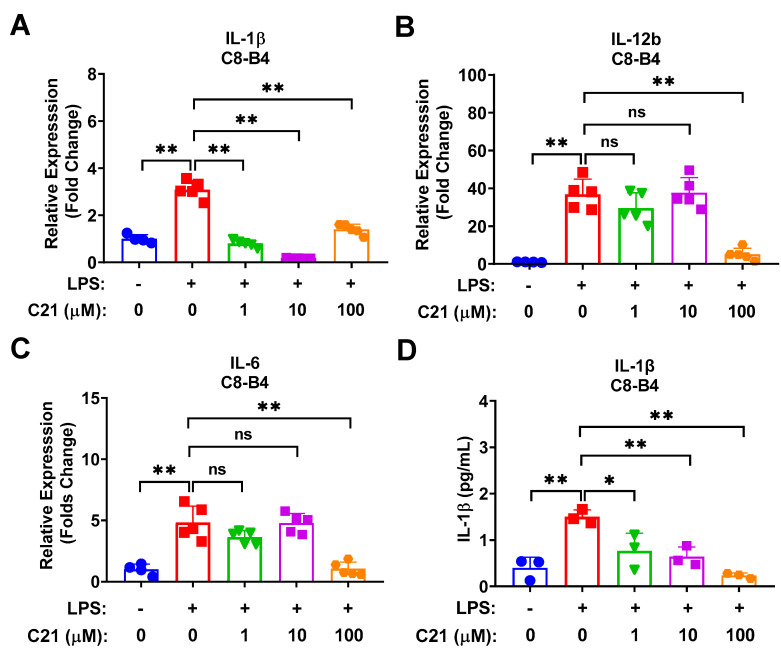
Compound **21** exhibits a profound anti-inflammatory response in microglia via decreasing the LPS-induced expression of pro-inflammatory cytokines/chemokines in a dose-dependent manner. C8-B4 mouse microglia cells were activated by LPS treatment (100 ng/mL) and co-treated with either a different concentration of C21 as indicated or the vehicle for 24 h. An amount of 500 ng of total RNA was reverse transcribed into complementary cDNA, and then gene expression was detected using SYBR Green-based RT-qPCR. PPIA was used as an endogenous control. As for protein expression, supernatants of cell culture medium were collected from the same experiment and centrifuged at 2000× *g* × 10 min for complete removal of cell debris and then the secreted cytokines/chemokines were assessed using ELISA kits. The mRNA expression level of pro-inflammatory cytokines and chemokines IL-1β (**A**), IL-12b (**B**), and IL-6 (**C**). The protein expression level of the pro-inflammatory cytokine, IL-1β (**D**). Values are the mean ± SD of 3–5 independent experiments analyzed by one-way ANOVA. ^ns^ *p* > 0.05, * *p* < 0.05, ** *p* < 0.01.

C8-B4 mouse microglial cells were co-cultured with LPS to induce the release of pro-inflammatory cytokines/chemokines, mimicking post-stroke inflammatory response, and treated with different concentrations of C21 (1 µM, 10 µM, and 100 µM). The LPS-mediated inflammatory response was evaluated at both the mRNA and protein levels. C21 demonstrated a dose-dependent reduction in the expression of pro-inflammatory cytokines/chemokines at the mRNA expression level, including IL-1β, IL-12b, and IL-6 (Figure 2A–C), and at the protein level of released IL-1β (Figure 2D).

### 3.3. Compound **21** Significantly Reduces the Expression of Pro-Inflammatory Cytokines/Chemokines in Macrophages in a Dose-Dependent Fashion

**Figure 3:**
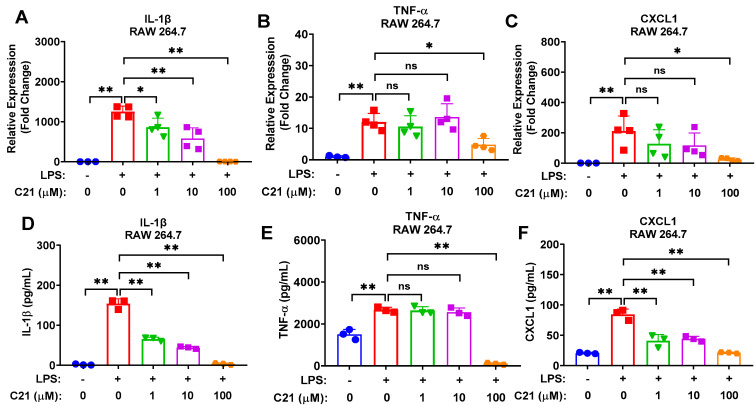
Compound **21** significantly ameliorates LPS-induced pro-inflammatory response (cytokine production) in macrophages in a dose-dependent manner. RAW 264.7 macrophage cells were cultured with LPS (100 ng/mL) and co-treated with different concentrations of C21, as indicated, or vehicle for 24 h. An amount of 500 ng of total RNA was reverse transcribed into complementary DNA, and then gene expression was detected using SYBR Green-based RT-qPCR. PPIA was used as an endogenous control. Protein expression was evaluated via ELISA. Cell culture supernatants were collected following a similar experimental design and centrifuged at 2000× *g* × 10 min for complete removal of cells’ debris, and then the secreted cytokines/chemokines were assessed. The mRNA expression level of pro-inflammatory cytokines and chemokines IL-1β (**A**), TNF-α (**B**), and CXCL1 (**C**). The protein expression level of pro-inflammatory cytokine/chemokines IL-1β (**D**), TNF-α (**E**), and CXCL1 (**F**). Values are the mean ± SD of 3–4 independent experiments analyzed by one-way ANOVA. ^ns^ *p* > 0.05, * *p* < 0.05, ** *p* < 0.01.

We examined the direct impact of C21 on the polarization status of RAW 264.7 macrophages. As illustrated in Figure 3, C21 significantly suppressed the expression of pro-inflammatory genes, IL-1β (Figure 3A), TNF-α (Figure 3B), and CXCL1 (Figure 3C), and pro-inflammatory proteins, IL-1β (Figure 3D), TNF-α (Figure 3E), and CXCL1 (Figure 3F), suggesting an anti-inflammatory effect of C21 via suppressing the release of pro-inflammatory cytokines/chemokines in both microglia and macrophages.

### 3.4. Compound **21** Effectively Regulates the Production of Reactive Oxygen Species (ROS) and Nitric Oxide (NO), and the Activity of Pro-Inflammatory Enzymes, iNOS, and COX-2, in Microglia/Macrophages

**Figure 4:**
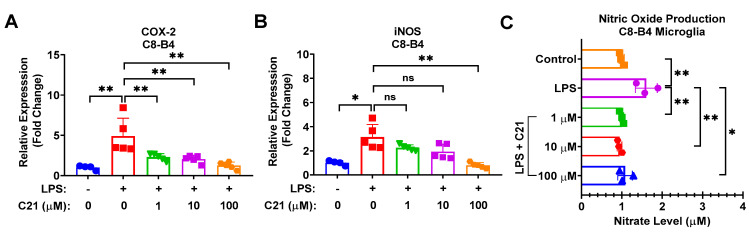
Compound **21** effectively regulates the production of NO and the activity of pro-inflammatory enzymes, iNOS and COX-2, in microglia. C8-B4 cells were incubated with LPS (100 ng/mL) and co-cultured with different concentrations of C21 as indicated for 24 h. The mRNA expression level of pro-inflammatory mediators, COX-2 (**A**) and iNOS (**B**). The Griess assay kit was used to quantify NO production. Cell culture supernatant was collected and centrifuged to eliminate cell debris, and then mixed with Griess reagent for 30 min at room temperature. The 548 nm absorbance was measured. Sodium nitrate was used as a reference. The level of nitrate (µM) was recorded using a microplate reader (**C**). Results are the mean ± SD of 3–5 independent experiments analyzed by one-way ANOVA. ^ns^ *p* > 0.05, * *p* < 0.05, ** *p* < 0.01.

**Figure 5:**
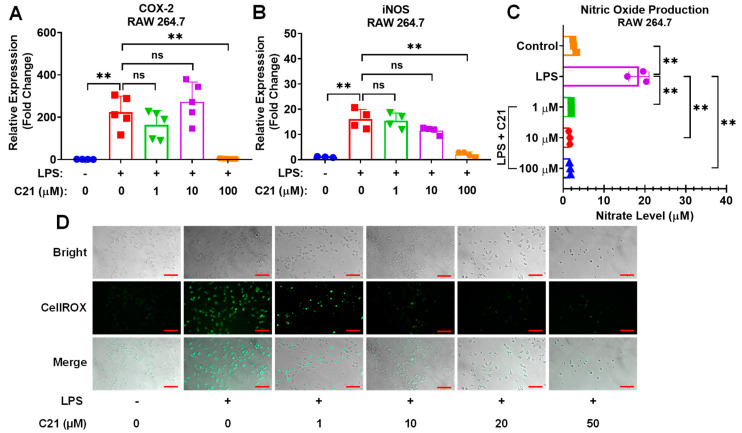
Compound **21** regulates the generation of ROS and NO, and the activity of pro-inflammatory enzymes, iNOS and COX-,2 in macrophages. RAW 264.7 macrophage cells were incubated with LPS (100 ng/mL) and/or different concentrations of C21, as indicated, for 24 h. An amount of 500 ng of total RNA was reverse transcribed into complementary DNA, and then gene expression was detected using SYBR Green-based RT-qPCR. PPIA was used as an endogenous control. The mRNA expression level of pro-inflammatory mediators COX-2 (**A**) and iNOS (**B**). Nitrate level (μM) was evaluated by Griess assay kit (**C**). After 24 h of treatment with LPS (100 ng/mL) and/or C21, the cell supernatants were centrifuged and incubated with Griess reagent. Absorbance was measured at 548 nm and sodium nitrate was used as a reference. For ROS production, cells were cultured with LPS (100 ng/mL) and co-treated with either different concentrations of C21 or vehicle for 24 h. Then, cells were washed with PBS and treated with 5 µM CellROXGreen Reagent for 30 min (**D**). Fluorescence images were obtained using a Zeiss Observer Z1 microscope (Carl Zeiss). Values are the mean ± SD of 3–5 independent experiments analyzed by one-way ANOVA. Scale bar, 100 μm. ^ns^ *p* > 0.05, ** *p* < 0.01.

As demonstrated in Figure 4 and Figure 5, C21 showed a profound effect in neutralizing the expression of COX-2 and iNOS in C8-B4 microglia (Figure 4A,B) and RAW 264.7 macrophages (Figure 5A,B), as well as in regulating the generation of free radicals, ROS and NO, production in both microglia (Figure 4C) and macrophages (Figure 5C,D). Our observations demonstrate that C21 normalizes the release of pro-inflammatory mediators and reduces the production of reactive oxygen species and other free radicals.

### 3.5. Compound **21** Is Associated with Increased Neuroprotective Activity in Microglia/Macrophages

**Figure 6:**
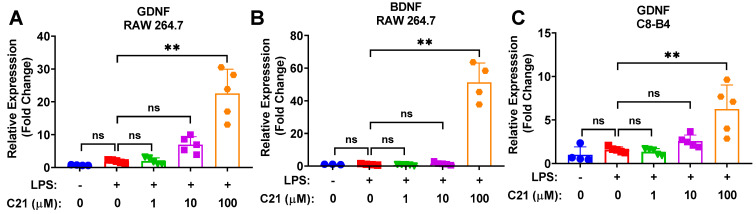
Compound **21** promotes the expression of neurotrophic growth factors (BDNF and GDNF) in a dose-dependent manner. RAW 264.7 macrophage and C8-B4 microglial cells were incubated with LPS (100 ng/mL) +/− C21 for a total of 24 h. mRNA expression levels of neurotrophic factors were analyzed using SYBR Green-based RT-qPCR. PPIA was used as an endogenous control. The mRNA expression level of neurotrophic factors GDNF (**A**) and BDNF (**B**) in RAW 264.7 macrophages (**A**,**B**), and of GDNF in C8-B4 microglia (**C**). Values are the mean ± SD of 5 independent experiments analyzed by one-way ANOVA. ^ns^ *p* > 0.05, ** *p* < 0.01.

We sought to investigate whether C21 increases the level of BDNF and other neurotrophic molecules via its direct effect on microglia and macrophages. Figure 6 demonstrates the neuroprotective effects of C21 in both microglia and macrophages, evaluated via the expression of neurotrophic factors, BDNF and GDNF. C21 demonstrated a dose-dependent upregulation of GDNF in C8-B4 microglia (Figure 6C) and an increase in the expression of GDNF and BDNF in RAW 264.7 macrophages (Figure 6A,B). This potentially indicates that C21 may play a neuroprotective role after stroke, which is mediated by the upregulation of neurotrophic factors both centrally and peripherally.

## 4. Discussion

After a stroke, microglia follow biphasic activation throughout the neuro-inflammatory response [26]. There is a transient activation of the anti-inflammatory phenotype followed by the activation of the M1-like phenotype [26]. However, during the delayed phase of neuro-inflammation, microglia are designed to switch back to the anti-inflammatory phenotype to facilitate long-term functional recovery, BBB repair, neurogenesis, and angiogenesis [4]. Similarly, in the early stage after stroke, the newly recruited macrophages assume the anti-inflammatory phenotype and gradually change to an M1-like phenotype [26,27]. Modulating microglia/macrophage activation after stroke toward an M2-like phenotype would be beneficial in modulating the inflammatory response and, ultimately, would facilitate long-term functional recovery.

The role of RAS modulation after stroke has been investigated by our group, and others, and investigations have demonstrated that Compound **21** (C21), the first selective non-peptide angiotensin II type 2 receptor agonist, provides a neurovascular protective effect and enhances sustained functional improvement at seven days after stroke. A single dose of C21 could reduce the infarct size and enhance the behavioral outcome without affecting blood pressure [17]. Moreover, RAS modulation with either C21 or Candesartan, an angiotensin receptor blocker (ARB), showed favorable outcomes regarding PSCI [21]. Post-stroke chronic administration of RAS modulators prevented the development of PSCI in hypertensive rats [14,19]. This effect was observed even with delayed administration of RAS modulators [14]. Along with preventing post-stroke cognitive decline, treatment with RAS modulators suppressed sustained microglial activation and prevented the microglial inflammatory response after stroke [14]. Also, the same findings were observed in diabetic animals, where C21 treatment was associated with improved functional recovery after stroke and a reduction in the inflammatory response, likely mediated through the modulation of microglial polarization (M1:M2 ratio) [22]. The M1:M2 ratio was upregulated in the ipsilateral hemisphere of diabetic animals after stroke, indicating a pro-inflammatory shift [22]. This was reversed after the delayed and long-term administration of C21 [22]. All things considered, these studies highlighted the importance of brain RAS and its modulatory effect on neuroinflammatory response as it emerges as a potential therapeutic target for PSCI. This study further supports these findings by showing the direct anti-inflammatory effects of C21 on microglia/macrophages in an in vitro setting.

**Figure 7:**
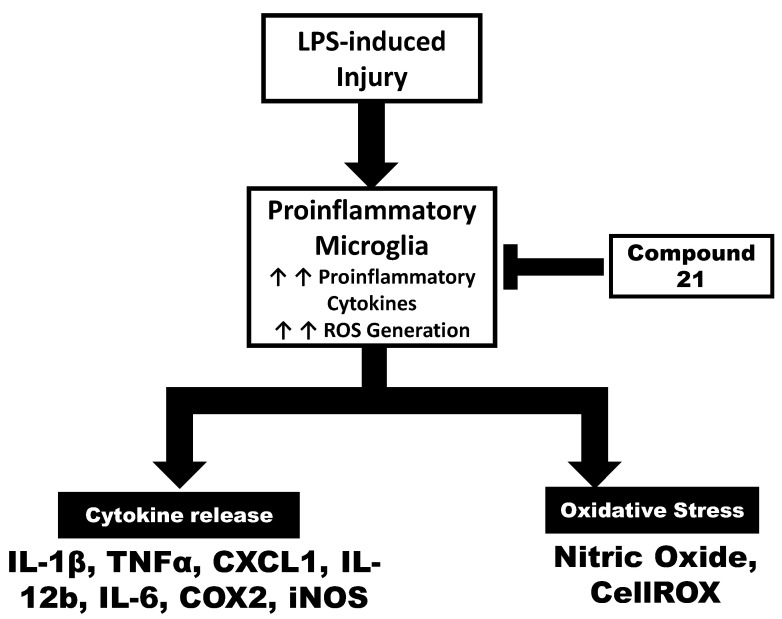
Schematic representation of the working hypothesis on Compound **21**-mediated effects on microglia and macrophages on oxidative stress and cytokine release as determined by ELISA and quantitative real-time rt-PCR analysis. ↑ upregulation.

In the current study, we demonstrated that the stimulation of AT2R, via C21, effectively modulates the polarization of microglia and macrophages toward a less pro-inflammatory phenotype. Our findings suggested that C21 exhibits an anti-inflammatory effect and reduces the expression of pro-inflammatory mediators in a dose-dependent manner, in both mouse microglial cells and RAW 264.7 macrophages. The expression of pro-inflammatory chemokines/cytokines was evaluated at both protein and mRNA levels. Furthermore, C21 was able to neutralize the expression level of iNOS and COX-2, as well as the generation of ROS, which has been shown to suppress post-stroke injury and mediate neuroprotective activities [28,29,30,31]. The current study provides additional insights into how C21 mediates its beneficial effects after stroke. As is evident from our study, C21 has dual anti-inflammatory and neurotrophic effects, affecting both microglia and monocyte-derived macrophages. C21 may stimulate the immune cells peripherally to produce neuroprotective/anti-inflammatory mediators, which in turn start a cascade of neuroprotective effects centrally. This could be facilitated via direct macrophage–microglia crosstalk or by stimulating macrophages to release neuroprotective molecules that can cross BBB and mediate its effects centrally (Figure 7).

Microglia and monocyte-derived macrophages share the same inflammatory response pattern and function. Both mediate an early pro-inflammatory response and release inflammatory cytokines/chemokines to cause further damage and exacerbate the ischemic injury during the acute phase of stroke [2,4,6,11,12]. Likewise, both cells modulate their phenotypic activation to shift the polarization status to facilitate an anti-inflammatory response and promote delayed recovery after stroke [2,4,6,11,12]. Few studies have looked at the direct interaction between microglia and macrophages, despite their functional and behavioral similarities in the brain [32]. A recent study revealed that macrophages directly downregulate the expression of pro-inflammatory genes in both mouse and human microglia [32]. LPS-stimulated BMDMs suppressed the expression of inflammatory genes in microglia, including IL-1β, TNF-α, and IL-10 [32]. Gene analysis of LPS-activated microglia in the presence of macrophages revealed that around 1076 genes were significantly differentially regulated, primarily those related to the NF–κB signaling pathway and apoptotic cell death. Keeping this in mind, our observations might indicate that the anti-inflammatory effects of C21 on macrophages could synergistically modulate the inflammatory status of microglia. We attempted to assess this by examining the impact of C21-treated macrophages on microglial inflammatory response using conditioned media concentrate; however, we could not reliably demonstrate such a concept due to experimental limitations.

The cytotoxic effect of C21 on microglia/macrophages is a major limitation of our study. C21 exhibited a slightly different pattern of toxicity on macrophages and microglia. In microglia, the highest two concentrations (50 and 100 μm) induced a significant reduction in cell viability, while in macrophages, only 100 μm did. This was confusing since some of the neurotrophic and anti-inflammatory effects were only observed at the highest concentration. In microglia, the positive effect of C21 on the expression of GDNF, iNOS, IL-12B, and IL-6 was only observed at 100 μm. In macrophages, a similar pattern was observed in the expression of TNF-α, CXCL1, COX-2, iNOS, GDNF, and BDNF. However, C21 still enhanced an anti-inflammatory effect in smaller concentrations within its cytoprotective range. This is evident in the expression levels of IL-1β, CXCL1, and COX-2, as well as in the nitric oxide production and the generation of ROS. These concentrations had been used in mouse macrophages and were shown to reduce nitric oxide production in a dose-dependent manner. The authors anticipated this to be unlikely related to the loss of viability [25]. A summary of cytokines and other genes that are modulated with C21 treatment in C8-B4 microglia and RAW 264.7 macrophages is provided in Table 1.

This controversy regarding the efficacy of C21 at different concentrations could be explained by the duration of treatment. Inflammatory cytokines peak at different time points and the ability of a compound to reverse this differs accordingly. The ability of smaller doses of C21 to modulate the inflammatory response was previously tested in THP-1 cells, following different treatment conditions, for 3, 6, and 12 h [33]. In that study, different patterns of reduction were observed at 3, 6, and 12 h [33]. Also, the trend at the mRNA level did not necessarily translate into protein expression across these time points [33]. For instance, C21 significantly regulated the mRNA expression of IL-6 at 3 h, but not at 6 and 12 h. Conversely, at the protein level, C21 significantly reduced the expression of IL-6 at 6 and 12 h, but not at 3 h [33]. Therefore, perhaps, the smaller concentrations of C21 we used (1 and 10 μm) induced an anti-inflammatory response at an earlier time point, and that could not be captured in our 24 h experiment.

To conclude, C21 exhibited a profound anti-inflammatory effect in microglia and macrophages via suppressing the mRNA/protein expression of pro-inflammatory mediators, as well as regulating the generation of free radicals that induce cellular stress. C21-mediated anti-inflammatory effects were associated with an increase in neurotrophic activity.

## Figures and Tables

**Table 1:** C21 suppresses the expression of LPS-induced pro-inflammatory response in microglia/macrophages and augments the neurotrophic effects.

	Microglia					Macrophages				
	Genes	LPS	LPS + C21 (1 μm)	LPS + C21 (10 μm)	LPS + C21 (100 μm)	Genes	LPS	LPS + C21 (1 μm)	LPS + C21 (10 μm)	LPS + C21 (100 μm)
mRNA	IL-1β	⬆⬆⬆	⬇⬇	⬇⬇⬇	⬇	IL-1β	⬆⬆⬆	⬇	⬇⬇	⬇⬇⬇
	IL-12B	⬆⬆⬆	No Change	No Change	⬇⬇⬇	CXCL1	⬆⬆⬆	No Change	No Change	⬇⬇⬇
	IL-6	⬆⬆⬆	No Change	No Change	⬇⬇⬇	TNF-α	⬆⬆⬆	No Change	No Change	⬇⬇
	COX-2	⬆⬆⬆	⬇⬇	⬇⬇	⬇⬇	COX-2	⬆⬆⬆	No Change	No Change	⬇⬇⬇
	iNOS	⬆⬆⬆	No Change	No Change	⬇⬇⬇	iNOS	⬆⬆⬆	No Change	No Change	⬇⬇⬇
	Gdnf	No Change	No Change	⬆⬆	⬆⬆⬆	Gdnf	No Change	No Change	No Change	⬆⬆⬆
						BDNF	No Change	No Change	No Change	⬆⬆⬆
Protein	IL-1β	⬆⬆⬆	⬇	⬇⬇	⬇⬇⬇	IL-1β	⬆⬆⬆	⬇	⬇⬇	⬇⬇⬇
						CXCL1	⬆⬆⬆	⬇⬇	⬇⬇	⬇⬇⬇

⬆ upregulation ⬇ downregulation.

## Data Availability

The raw data supporting the conclusions of the present study are available from the corresponding author upon request.

## Supplementary Materials

The following supporting information can be downloaded at: https://www.mdpi.com/article/10.3390/life13061274/s1, Figure S1: Standard curve plots of IL-1β, TNF-α, and CXCL1 ELISA. Table S1: Primer sequences of various genes used in the qRT-PCR analysis.
