# Supplementary material for: Stimulation of Angiotensin II Type 2 Receptor Modulates Pro-Inflammatory Response in Microglia and Macrophages: Therapeutic Implications for the Treatment of Stroke
Source: Life (Basel). 2023 May 29;13(6):1274. doi: 10.3390/life13061274 (PMC10302703; doi:10.3390/life13061274)
Supplement: Supplementary file 1 [file life-13-01274-s001.zip › life-2334333-supplementary/life-2334333-supplementary-Figure S1.pdf]

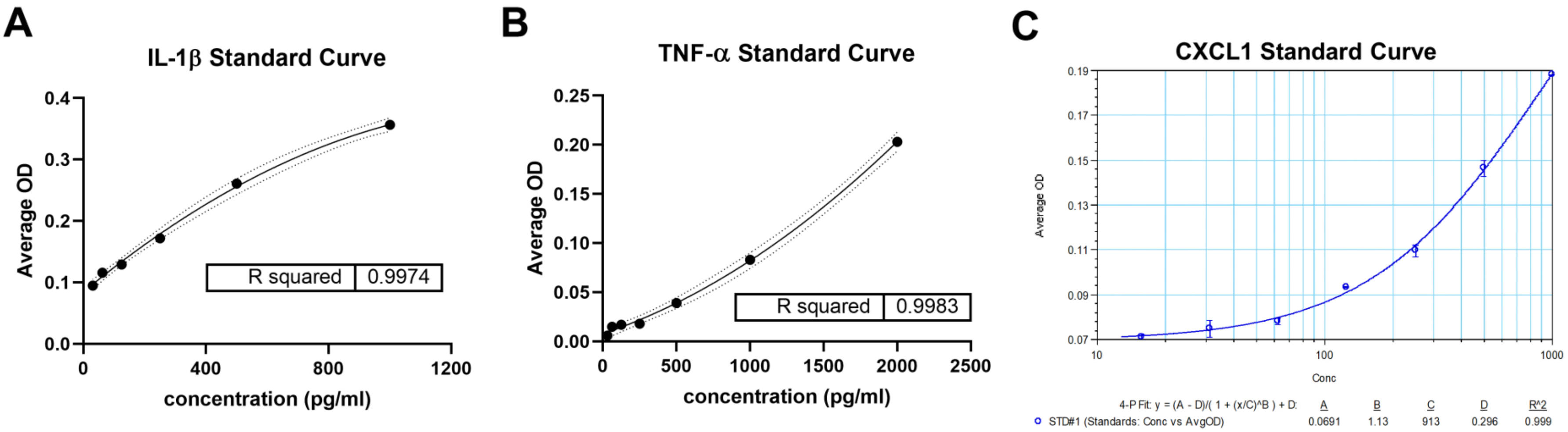

Supplemental Figure S1: Standard curve plots of IL-1β, TNF-α, and CXCL1 ELISA
