# Supplementary material for: Stimulation of Angiotensin II Type 2 Receptor Modulates Pro-Inflammatory Response in Microglia and Macrophages: Therapeutic Implications for the Treatment of Stroke
Source: Life (Basel). 2023 May 29;13(6):1274. doi: 10.3390/life13061274 (PMC10302703; doi:10.3390/life13061274)
Supplement: Supplementary file 1 [file life-13-01274-s001.zip › life-2334333-supplementary/life-2334333-supplementary-Table S1.pdf]

Supplemental Table S1: Primer sequences of various genes used in the qRT-PCR analysis.

GENE		Sequence
**BDNF**	m_BDNF_Forward	GCGCCCATGAAAGAAGTAAA
	m_BDNF_Reverse	TCGTCAGACCTCTCGAACCT
**GDNF**	m_GDNF_Forward	TGACTCCAATATGCCTGAAGATTATC
	m_GDNF_Reverse	AATGGTGGCTTGAATAAAATCCA
**IL-12B**	m_IL-12B_Forward	ACAGCACCAGCTTCTTCATCAG
	m_IL-12B_Reverse	TCTTCAAAGGCTTCATCTGCAA
**IL-1β**	m_IL-1β_Forward	TGGACCTTCCAGGATGAGGACA
	m_IL-1β_Reverse	GTTCATCTCGGAGCCTGTAGTG
**TNFα**	m_TNFα_Forward	CCCTCACACTCAGATCATCTTCT
	m_TNFα_Reverse	GCTACGACGTGGGCTACAG
**COX2**	m_COX2_Forward	TTCAACACACTCTATCACTGGC
	m_COX2_Reverse	AGAAGCGTTTGCGGTACTCAT
**NOS2**	m_NOS2_Forward	GGCAGCCTGTGAGACCTTTG
	m_NOS2_Reverse	GCATTGGAAGTGAAGCGTTTC
**IL-6**	m_IL-6_Forward	TAGTCCTTCCTACCCCAATTTCC
	m_IL-6_Reverse	TTGGTCCTTAGCCACTCCTTC
**CXCL1**	m_CXCL1_Forward	CTGGGATTCACCTCAAGAACATC
	m_CXCL1_Reverse	CAGGGTCAAGGCAAGCCTC
**PPIA**	m_PPIA_Forward	GAGCTGTTTGCAGACAAAGTTC
	m_PPIA_Reverse	CCCTGGCACATGAATCCTGG
